# Monoallelic Gene Expression: Stochastic or Clonal? From Detection to Mechanisms and Clinical Significance

**DOI:** 10.3390/ijms27146262

**Published:** 2026-07-14

**Authors:** Olga A. Zemlianaia, Vladimir V. Strelnikov, Dmitry V. Zaletaev, Marina V. Nemtsova

**Affiliations:** 1 Research Centre for Medical Genetics, 115522 Moscow, Russia; zalnem@mail.ru (D.V.Z.); nemtsova_m_v@mail.ru (M.V.N.); 2 Department of Translational Medicine and Biotechnology, Sechenov University, 119991 Moscow, Russia

**Keywords:** monoallelic expression, DNA methylation, histone modifications

## Abstract

There are several forms of monoallelic expression, in which the 50:50 ratio in the expression activity of each of the two copies of the gene is disrupted. This review focuses on the phenomenon of random autosomal monoallelic expression (aRME), a process in which only one of the two alleles is transcribed in a single cell, and the allele choice is not determined by the parental origin. One of the central problems in studying this phenomenon is the differentiation of the two forms of aRME: clonal, in which the expression pattern is mitotically inherited, and stochastic, when the allele choice can change over time due to transcriptional bursting. Distinguishing these events is methodologically challenging: divergent experimental approaches and the lack of standardized detection criteria have produced strikingly contradictory estimates of aRME prevalence. Furthermore, single-cell transcriptomic approaches are particularly susceptible to technical artefacts that can generate false-positive monoallelic calls, complicating the distinction between heritable and transient states. In this review, we critically evaluate the evidence for clonal and stochastic aRME, examine the epigenetic mechanisms proposed to maintain monoallelic expression, and discuss its clinical significance, focusing on the role of monoallelic expression in the penetrance of autosomal dominant diseases (including congenital immune disorders and cardiomyopathy), neurodevelopmental disorders, and cancer progression.

## 1. Introduction

The diploidy paradigm implies that we inherit a complete set of chromosomes from each parent. Among other benefits, this arrangement creates genetic variation via homologous recombination and provides certain protection against deleterious mutations, as a functional product translated from the unaffected allele can often compensate for a defective copy from the mutant allele [1]. It is generally believed that two copies of each gene are required to achieve the desired level of product, which would allow proper regulation of the developmental processes, from cellular differentiation to organ formation. Indeed, for the vast majority of genes in mammalian cells, both alleles are transcribed, contributing to the pool of gene expression. However, many exceptions are known to this day, which prove that the precise dosage of gene product can be maintained via strict monoallelic expression (a complete silencing of one allele) or allelic imbalance (AI) (a strong deviation from a 50:50 ratio in allelic expression) [2,3].

Today, several mechanisms are known that are used by eukaryotic cells to suppress the expression of one allele, among which the most common one is epigenetic regulation. Epigenetic mechanisms regulate gene expression without directly influencing DNA sequence; instead, they act via spatial influences that affect DNA accessibility to transcription factors and other regulatory molecules. Epigenetic mechanisms include DNA methylation/demethylation, histone protein modifications, chromatin remodeling, and the action of noncoding RNAs. Acting in a complex manner, all of these mechanisms, and particularly histone modifications and DNA methylation, alter the accessibility of genes to transcription factors providing stable but reversible marks that influence gene expression [4]. Histone proteins (core histones H2A, H2B, H3, and H4, which form an octamer, and linker histone H1 which binds nucleosomes) undergo numerous post-translational modifications including methylation, acetylation, phosphorylation, ubiquitination, sumoylation, glycosylation, and poly-ADP-ribosylation. The effect of certain modification on gene expression depends not only on its type, but also on the amino acid position and the number of groups attached to it [5,6] DNA methylation is a covalent modification of cytosine bases, primarily in CpG dinucleotides, which typically functions as a robust silencing tool, recruiting proteins that condense chromatin and make it inaccessible to the transcriptional machinery, thereby ensuring genes are expressed only in the correct cellular and developmental contexts [7,8].

One of the most recognizable examples of the epigenetically driven allele choice is genomic imprinting. The phenomenon of genomic imprinting suggests that each copy of an imprinted gene is expressed or repressed depending on its parental origin. In humans, more than 250 genes are known to be subject to imprinting [9]. Another deviation from the diploid expression paradigm occurs through the phenomenon known as random monoallelic expression (RME). In RME, a gene is transcribed from only one allele in a cell, and this pattern can be either clonally propagated (clonal RME) or emerge in a dynamic, rapidly changing manner due to transcriptional bursting (stochastic RME) [10]. The primary example of epigenetically driven clonal RME is random X-chromosome inactivation (XCI) in female mammals which is established in all cells in a random manner at the early stage of embryogenesis and is propagated during mitotic cell divisions throughout life. Although X-chromosome inactivation is the most noticeable example of gene dosage compensation via random allele exclusion, extensive evidence suggests that autosomal monoallelic expression (aRME) exists and is detected in a subset of autosomal genes. While both processes generate cellular mosaicism through random monoallelic expression, they are fundamentally distinct. XCI is a chromosome-wide dosage compensation mechanism governed by the master regulator (the *Xist* long non-coding RNA), ensuring stability across tissues. In contrast, clonal aRME involves individual genes scattered across autosomes, lacks a singular master regulator, and may be observed in a limited number of cells. Clonally propagated autosomal monoallelic expression provides a powerful explanation for certain puzzling genetic observations. For instance, it can underlie unusual inheritance patterns where a disease trait appears to «skip» generations or affect individuals unpredictably. It can also serve as a primary contributor to incomplete penetrance—the phenomenon where not all individuals carrying a dominant disease-causing mutation develop the associated phenotype [11].

This review focuses on the existing knowledge about the prevalence of aRME in various tissues, the putative epigenetic mechanisms underlying this phenomenon, and its biological significance in the context of human diseases. We searched for the literature in the PubMed and Google Scholar scientific databases. The search strategy included combinations of keywords: «Random monoallelic expression», «Allelic skewing», «Monoallelic DNA methylation», «Allelic imbalance» AND «Epigenetic regulation», «Allele-specific methylation», «Monoallelic expression in disease». The publication period was 20 years; special attention was paid to the studies conducted in the last 5 years. Priority was given to reviews and original research in peer-reviewed scientific journals. An additional search was conducted using the reference lists from the found articles, as well as among the works of authors who have published more than two scientific papers on the topic.

## 2. Random Autosomal Monoallelic Expression: Clonal vs. Stochastic

It is known that various forms of monoallelic expression are necessary for specific stages of development; this includes gene dosage compensation, which is essential for female mammals through the inactivation of one of the X chromosomes. Once established early in female development in individual cells through random selection, the pattern of monoallelic X-chromosome gene expression is stably maintained through mitosis.

One of the first groups of autosomal genes to be recognized as monoallelically expressed was the olfactory receptor gene family. The study by A. Chess et al., which was based on the assumption that olfactory receptor genes are subject to random monoallelic expression, was of great significance as it demonstrated for the first time that only one of the two copies of an olfactory receptor gene is active, and this process does not require obligatory DNA rearrangements [12]. The expression of a single copy of the receptor by individual neurons ensures functional differentiation and specificity in odor recognition. In contrast to the genomic imprinting mechanism which had already been established at that time, the 1994 study revealed that in the case of odorant receptor genes the parental legacy of the expressed allele is random, implying that half of the cells express the maternal copy of the gene, and the other half express the paternal copy.

A few years later, monoallelic expression was also demonstrated for a number of genes encoding cytokines in T-cells, underlying that random allele choice determines the diversity of the combinations of regulatory molecules produced by antigen-specific cells [13,14,15,16,17]. A similar expression pattern was established for the interferon-beta gene *IFN-β* at the earlier stages of induction [18]. Viral infection triggers its stochastic expression from a single allele in a subset of cells, and allelic choice was shown to depend on the interchromosomal associations of *IFN-β* with at least one of the three genomic loci that help to deliver a limiting transcription factor (NF-κB) to the enhancer, enabling enhanceosome assembly. The IFN-β protein produced then acts as a signal amplifier by inducing IRF-7, which in turn promotes enhanceosome assembly and IFN-β transcription from the remaining alleles and in other cells. Another study investigating monoallelic expression of autosomal genes described a unique RME pattern for the protocadherin-α (*Pcdha*) gene cluster in individual mouse neurons. The vertebrate brain expresses a variety of protocadherin-α molecules, also known as neuronal cadherin-associated receptors. Their genomic organization includes multiple variable exons and a set of constant exons, similar to the immunoglobulin (Ig) and T-cell receptor genes. The authors analyzed allelic expression of the *Pcdha* gene cluster in individual neurons and demonstrated monoallelic and combinatorial expression of each variable exon in the *Pcdha* genes. Each variable exon of *Pcdha* was transcribed monoallelically, but a single neuron could simultaneously express multiple different exons from both parental chromosomes, thus forming combinatorial sets of transcripts to provide a wide range of adhesive properties for individual neurons [19]. These results prompted further research to elucidate the influence of aRME on the formation of phenotypic diversity.

Monoallelic expression analysis is largely based on the integration of transcriptomic and genomic data. In order to distinguish between the two alleles, the mRNA assay must be paired with DNA genotyping data for the same individual. This DNA data is essential to identify informative heterozygous single nucleotide polymorphisms (SNPs)—positions which allow for the two alleles to be recognized as maternal and paternal. One of the first attempts to explore the genome-wide distribution of autosomal monoallelically expressed genes was in 2007, when Gimelbrant et al. used modified Affymetrix SNP arrays on clonal human cell lines to assess allele-specific transcription of 4000 genes in B-lymphoblastoid cell lines [20]. Given the existing knowledge about X-inactivation, the study took the probable clonal nature of ME propagation into consideration; thus, clonal cell lines were derived using single-cell cloning. The researchers found that more than 300 (>5%) of the genes studied exhibited monoallelic expression based on the SNP analysis data. This can lead to differences in protein repertoires and differences in gene expression levels. Thus, the authors concluded that widespread monoallelic expression utilizes a mechanism that generates diversity in individual cells and their clonal progeny. Despite numerous limitations of SNP-based analysis, including the relatively small number of heterozygous exonic SNPs and uneven representation of different genes in the transcriptome, new studies adapting this method rapidly emerged, particularly with a focus on aRME in the central nervous system [21,22]. In another study, monoallelic expression patterns were studied in the mouse brain transcriptome using SNP analysis of 122 candidate genes in six independent F1 hybrid clonal neural stem cell (NSC) lines. Three genes (*Camk2a*, *Kcnc4*, *Unc5a*) showed ≥90% expression of the same parental allele in all six NSC lines, while six other monoallelic genes exhibited heterogeneous patterns. Some clones expressed only one allele, while others demonstrated biallelic expression or a predominance of the opposite allele. The results indicate that 1–2% of genes expressed in the CNS may be subject to allelic exclusion; remarkably, some of these genes are involved in neurodevelopment and are associated with various CNS diseases [22]. These findings once again reinforced the interest of scientists in searching for aRME in different tissues at different stages of cell proliferation.

The paradigm of aRME being a widespread, clonally propagated feature of numerous genes shifted in 2014. In the study by Deng et al., 269 individual cells were derived from in vivo embryos from oocyte to blastocyst stages of mouse preimplantation development [23]. The authors found significant (12–24%) monoallelic expression of autosomal genes, with the choice of allele for expression occurring independently. Surprisingly, monoallelic expression appeared to be random and dynamic, as significant variations were observed in closely related embryonic cells. Pooling cells from the same embryo eliminated the monoallelic signal, indicating that the expression pattern is cell-specific and random phenomenon. Similar patterns of monoallelic expression were subsequently observed in mature cells. It was concluded that independent and stochastic allelic transcription generates random monoallelic expression in mammalian cells, casting doubt on the idea of clonal propagation of aRME [23]. The comparison of the expression levels revealed that genes expressed biallelically produce approximately twice as much RNA as the same genes expressed monoallelically, consistent with the expected transcriptional activity of each allele. This two-fold difference supports a model in which stochastic monoallelic expression causes independent increases in transcriptional activity at each allele [23].

In accordance with those observations, the highly stochastic and dynamic nature of aRME in fibroblasts was demonstrated in the Borel et al. study [24]. The authors used single-cell RNA sequencing to determine mRNA levels and specific alleles in 203 individual primary human fibroblasts for 133,633 unique heterozygous single-nucleotide variants (hetSNVs). They found that at the time of analysis, each cell contained predominantly transcripts of one allele of most genes: approximately 76.4% of hetSNVs exhibited stochastic monoallelic expression in individual cells. Remarkably, closely spaced hetSNVs formed a haplotype, while distant SNPs located in two different genes were independent of haplotype structure. For most actively transcribed genes, the results showed that one allele was predominantly detected in a given cell at a given time, while the other allele was present at low levels or undetectable. Interestingly, only a few genes were found with identical mRNA levels of both alleles in all individual cells. This study characterized allele-specific transcription of human autosomal genes as a highly dynamic and stochastic process that has direct implications for cellular phenotypic variability.

**Figure 1:**
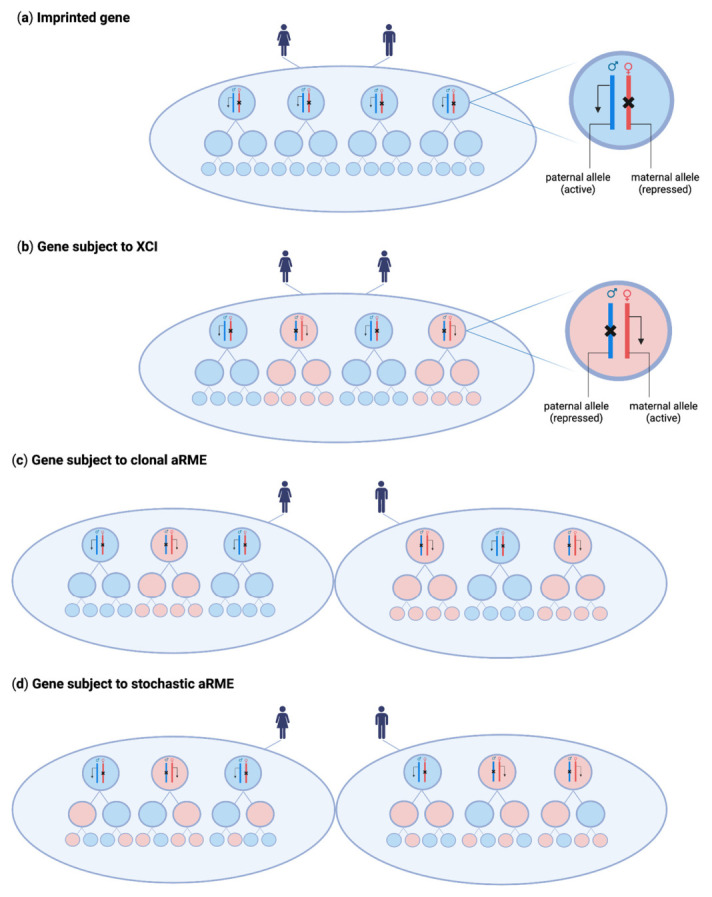
Different patterns of monoallelic expression and the resulting cell populations. (**a**) An imprinted gene is expressed from the allele of a specific parental origin in all individuals, resulting in a homogeneous cell population. (**b**) A gene located on the X chromosome in females undergoes random monoallelic expression in every cell of the organism. The cell population is heterogeneous, with each allele contributing roughly 50% to the overall expression pool in all female individuals. (**c**) A gene undergoing random autosomal monoallelic expression that spreads clonally can be expressed from different alleles in different cell populations. The pattern of allelic expression may differ among individuals. (**d**) A gene undergoing stochastic random autosomal monoallelic expression is expressed from different alleles at any given moment. The cell population is heterogeneous, and the pattern of allelic expression differs among individuals. Created in https://BioRender.com.

As a result of the conducted studies, it became clear that monoallelic expression can have a profoundly variable molecular background, which severely complicates its detection: stochastic and clonal monoallelic expression have a similar allelic expression pattern in regard to the resulting cell population (Figure 1). The main difference between clonal and stochastic monoallelic expression lies in their molecular basis. Stochastic aRME refers to allelic expression that is transient and essentially not mitotically transmitted between cell divisions. It requires no stable maintaining mechanisms; instead, it is explained by the intrinsic stochasticity of allelic expression. Transcriptional noise arises from both the probabilistic switching of gene activity and the variation in RNA output per active period, known as transcriptional bursting. Both alleles have an equal probability of initiating transcription, and the exact timing of allelic bursts is stochastic. The resulting episodic allelic output produces periods where the expression in a cell is monoallelic, biallelic, or absent entirely. Presumably, a gene’s transcription bursting frequency, molecules per burst, and RNA degradation rate determine the probability of monoallelic or biallelic expression at any given time [25]. Thus, in contrast to the clonal aRME, the allelic expression switches between divisions rather than being inherited.

In the following years, a significant difference between the abundance of clonal and stochastic aRME in the mouse and human genomes was demonstrated. Reinius et al. used allele-sensitive RNA-seq on primary mouse CD8^+^ fibroblasts and human T-cells in vivo to determine whether random monoallelic expression of autosomal genes (aRME) is mitotically inherited (clonal) or stochastic (dynamic) in somatic cells [26]. This study utilized single-cell transcriptomics to analyze the nature of monoallelic expression in somatic cells, providing the first genome-wide, allele-sensitive single-cell measurement of aRME in primary human immune cells in vivo. After clonally expanding individual mouse primary fibroblasts and tracking the CD8^+^ T-cells during the acute and memory phases of the vaccine response, the authors measured aggregated monoallelic calls for each clone as well as for non-clonal cells and computed the fraction of genes that were consistently monoallelically expressed. Importantly, the study used a threshold of 98% for the number of reads aligned to a single allele to consider expression monoallelic. Overall, the authors observed monoallelic expression for 13% of autosomal genes in fibroblasts among highly expressed genes (RPKM > 20), while the analysis including all expressed genes (RPKM > 1) revealed that the frequency of clonally expanding aRME events was only 0.5%. As for CD8^+^ T-cells, aRME was observed for ~60–85% of expressed genes (RPKM > 20), while clonal aRME was only observed for 0.9% of genes. These results challenge the previously established paradigm of widespread clonal aRME affecting thousands of genes and demonstrate that the vast majority of cellular variation in allelic expression occurs randomly across somatic cell populations and fluctuates over time. Moreover, it suggests that clonal aRME, when present, tends to affect low-expressed genes. Further studies revealed that the reason for the detection of clonal aRME only for low-expressed genes was the misidentification of monoallelic expression due to stochastic and dynamic transcription [27].

As the results regarding the abundance of genes undergoing clonal and stochastic aRME differed significantly (Table 1), an open discussion of this process took place in 2018 [28,29]. The discrepancies between the studies may occur due to different requirements for monoallelic expression of genes and the different methodologies used to study aRME. Naturally, earlier studies demonstrating the prevalence of aRME used lower standards for expression level deviation. Different researchers impose different requirements for the expression level and the degree of asymmetry required to consider an event a true manifestation of aRME. Even greater variation is observed in the criteria for allelic proportion: some authors utilize a threshold of 70:30 (meaning expression can be considered monoallelic if one allele accounts for at least 70% of the mRNA), while others use 80:20 or 90:10, given that the sequencing coverage is sufficient. Therefore, the same dataset would contain thousands of aRME loci when analyzed according to the criteria of some researchers and only dozens while using the criteria of others. Furthermore, it is important to note that differences in results may arise when analyzing aRME patterns using immortalized cell lines and in vivo, as stochastic genomic changes occurring in the former scenario may affect the differentiation process [29].

Apart from the different study designs and expression requirements, the monoallelic gene expression analysis using RNA-seq encompasses several bioinformatic challenges which can contribute to the low reproducibility of the experimental data. After establishing informative SNPs, the analytical pipeline proceeds to allele-specific read alignment and counting using specific aligning tools. This step is crucial for obtaining the accurate information on the monoallelic expression rate, as standard aligners are prone to reference allele bias, systematically favoring reads that match the reference genome and causing false monoallelic signal detection. The analysis becomes considerably more complicated when working with single-cell data or genes that are not captured well by standard RNA-seq. In scRNA-seq experiments, an allele may escape detection not because it is silenced, but because the starting RNA amount is insufficient for effective reverse transcription. Another obstacle comes from the fact that RME analysis requires read counts to be combined in a phase-aware manner. That is, for short reads that assess individual heterozygous SNPs, allelic counts must be summed according to the parental chromosome of origin across all informative sites within a given transcript. Naturally, the relative phasing of sites is often not known with certainty from the available sequencing data [30]. Furthermore, as it was previously indicated, a large proportion of genes lack a sufficient number of exonic informative SNPs, preventing allelic assessment altogether, regardless of their transcriptional output.

**Table 1:** Autosomal random monoallelic expression in various tissues.

Link to the Study	Cell/Tissue Type	Number of Clones Studied	Number of Genes Studied	Number of Genes Undergoing aRME
Neural tissue				
[23]	Mouse neural stem cells	4 clones	7198	∼140 (2%)
[22]	Mouse neural stem cells	6 clones	122	9 (7%)
[31]	Human neural stem cells	9 clones obtained from 3 individuals	~9000	128–169 depending on the part of the brain (~1.5%)
[32]	Mouse neural progenitor cells (NPC) derived from embryonic stem cells (ESCs) in vitro; Mouse astrocytes	8 NPC clones; Astrocytes from 2 NPC clones	14,415	394 (3%)
[33]	Mouse NPCs	249 NPC clones	12 genes for which aRME was previously demonstrated	Allelic expression imbalance was shown for all the studied genes
Embryonic tissues				
[34]	Mouse ESCs and neural progenitor cells obtained in vitro	6 clones (from both ESCs and induced NPCs)	13,699	ESCs: 67 (0.5%) NPCs: 376 (3%)
[23]	Cells at different stages of mouse preimplantation development (from oocyte to blastocyst)	269 single cells	~13,000	~1560–3120 (12–24%)
Hematopoietic tissues				
[20]	Human B-lymphoblastoid cell lines	Multiple clones obtained from 3 individuals	~4000	>300 (8%)
[26]	CD8^+^ Human T-cells (differentiation in vivo)	9 clones	806	Total aRME: ~60–85% Clonal aRME: 0.9%
[35]	Mouse hematopoietic stem cells (HSCs); CD19^+^ IgM^+^ B cells, CD4^+^ CD8^+^ mouse T-cells (after in vivo differentiation)	B-cells: 5 clones; T-cells: 2 clones	Complete autosomal transcriptome	14 (clonal aRME)
Stromal tissues				
[24]	Human primary fibroblasts	203 single cells from two different cell lines of human primary fibroblasts	568	35 (6%)
[26]	Primary mouse fibroblasts	7 clones	10,702	Total aRME (RPKM > 20): 13% Clonal aRME (RPKM > 1): 0.5%
Bulk tissue analysis				
[36]	54 tissues	-	~22,000	2762 (12.5%)

The actual share of stable clonally propagated aRME was once again questioned in the in vivo study by Kubasova et al. [35]. The authors transplanted single, highly heterozygous donor hematopoietic stem cells (HSC) into recipient mice and analyzed allele-specific transcriptomes of B- and T-cells derived after 12 weeks of extensive differentiation. In contrast with the previous belief that the allelic transcription patterns could be established during differentiation stages and clonally propagated in the process [32,37], the study showed that highly differentiated cells carry virtually no allelic transcriptional deviations. Only a tiny subset of autosomal genes (<0.2%) was identified whose allelic expression bias was already set in the transplanted HSC and remained through extensive differentiation to B- and T-cell lineages, suggesting that aRME patterns could be progressively erased and reestablished along the differentiation steps, at least in HSCs.

The dynamic nature of gene expression prompted a reconsideration of the aRME concept and introduced random allelic expression imbalance (RAExI), which implies the existence of several stable states of allelic expression per gene [33]. In the 2021 study, the authors analyzed over 200 neural progenitor cell (NPC) clones and found that 12 previously identified aRME genes exhibit distinct expression patterns, allowing them to be classified into allelic expression imbalance subtypes. The study demonstrated that genes previously classified as aRME may in fact be expressed in one or more subpopulations of clones with different allelic ratios, ranging from highly monoallelic to slightly biased or completely biallelic expression. Moreover, these modalities were suggested to be established and maintained during the transition from ESCs to NPCs, as allelic expression ratios for the genes studied were stable after differentiation.

One of the recent studies attempted to detect both clonal and dynamic aRME genes by comparing relative expression of the two alleles across different tissues using RNA-seq datasets from a single person [36]. In this study, the authors developed and demonstrated a method for profiling aRME in human tissues to identify highly reliable genes with random allelic and biallelic expression. The authors imply that if a gene is expressed from both alleles or expressed from only one allele in all cells due to genomic imprinting effects or *cis-* genetic events, the expression variation among different cell types would be low. However, if a gene demonstrates random mono-allelic expression (either autosomal or X-linked), the variation would be much higher. According to this, approximately 10% of autosomal genes in adult men and women were shown to exhibit aRME. The study identified 2762 genes with a high degree of random allelic expression across all major human tissues, and 1985 in the brain. These genes exhibit substantial aRME within individuals, are commonly found across the population cohort, and demonstrate reproducibility between males and females. Crucially, high-confidence aRME genes were shown to be enriched in rapidly evolving genomic regions, adaptive signaling processes, and significantly linked to age-related diseases like cancer and neurodegeneration.

**Figure 2:**
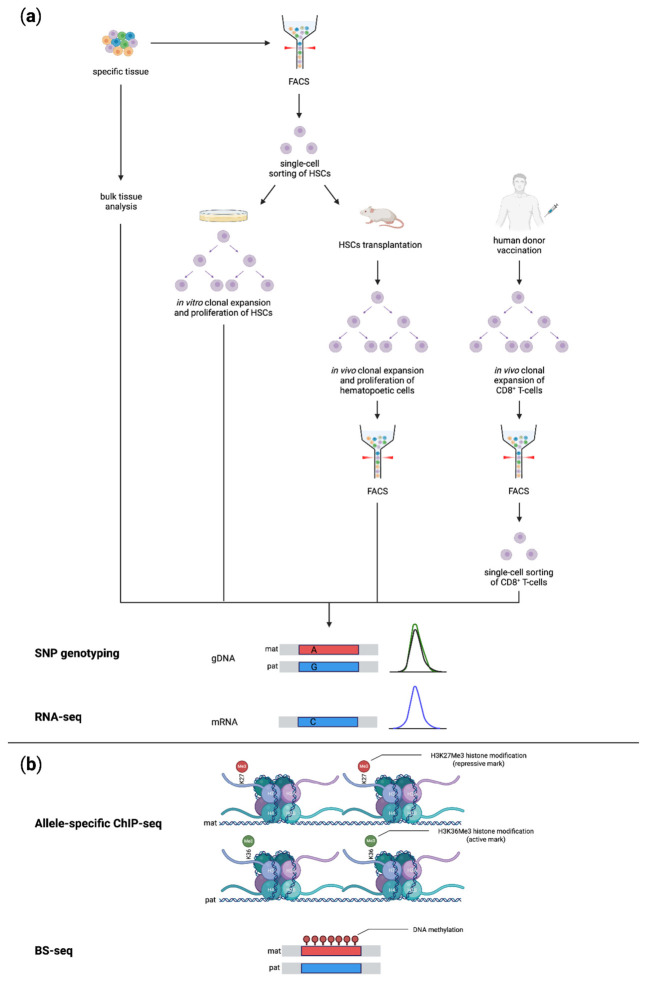
Approaches to studying monoallelic expression using hematopoietic cells as an example. (**a**) Genes undergoing autosomal random monoallelic expression can be searched for in whole tissue, in cell clones obtained by in vitro differentiation, and in cell clones obtained by in vivo differentiation (as demonstrated by Kravitz (2023) [36], Kubasova (2022) [35], and Reinius (2016) [26]). Identification of monoallelic expression of genes can be performed at the mRNA level by single-nucleotide polymorphism (SNP) genotyping and subsequent RNA-seq. The blue peak represents cytosine, the green and the black peaks represent adenine and guanine, respectively. mat, maternal allele (red-colored); pat, paternal allele (blue-colored). (**b**) Identification of monoallelic expression of genes can be performed by determining allele-specific histone modifications and monoallelic DNA methylation. Created in https://BioRender.com.

According to the literature presented, the study of aRME by various groups of scientists has led to the development of a large number of approaches to the search for monoallelic genes (Figure 2a). Overall, it is clear that aRME is a widespread mechanism for regulating gene expression; however, the problem of distinguishing between stochastic and clonal aRME remains relevant due to their fundamentally different biological significance. If a gene undergoes clonal aRME, any loss-of-function genetic variation in that allele can result in the complete absence of expression or a significant reduction in expression, increasing susceptibility to diseases caused by haploinsufficiency. Similarly, if the active allele carries a dominant-negative or gain-of-function mutation, monoallelic expression can enhance its pathogenic effects, since the silent allele cannot mitigate this effect.

Accurate identification of the genes undergoing clonal aRME requires an integrated understanding of the multiple regulatory layers that can establish and maintain this process. From the genetic standpoint, the presence of cis-acting sequence variants (such as SNPs in promoter or enhancer regions) can directly influence transcription factor binding affinity, thereby creating a constitutive bias toward one allele. However, genetic variation alone is insufficient to explain the stochastic or clonal patterns observed in genetically identical cells. The variability of the allelic choice may arise at the transcriptional level, leading to the fundamental problem of distinguishing between clonal and stochastic aRME using RNA analysis. The primary source of confusion when attempting to identify truly random monoallelic expression is the highly dynamic nature of transcription. In clonal aRME, these transcriptional dynamics are thought to be fixed by epigenetic marks, converting a stochastic event into a heritable, deterministic pattern. Thus, epigenetic regulation is the most widespread mechanism to investigate in the context of clonal aRME.

## 3. Can Epigenetic Regulation Actually Determine Stable aRME?

### 3.1. Epigenetic Events Driving Monoallelic Expression in X Inactivation and Genomic Imprinting

The transcriptional state of a large number of genes is known to be regulated by epigenetic mechanisms, including DNA methylation, post-translational modifications of histones, ATP-dependent chromatin remodeling, and non-coding RNAs. As we have already mentioned above, the influence of a particular epigenetic modification on gene expression depends not only on the type of chemical group attached but also on the substrate and the position at which the modification occurs (Table 2).

The recruitment of chromatin-modifying enzymes responsible for establishing specific histone marks can occur through specific DNA-binding transcription factors, as well as through DNA methylation patterns. Furthermore, histone modifications are involved in targeting DNA methyltransferases (DNMTs) to certain DNA loci. For instance, the euchromatin-specific modification H3K4me3 impairs the ability of DNMT3A, DNMT3B, and DNMT3L to bind to the N-terminal tail of histone H3 and thus prevents DNA methylation, which promotes chromatin decondensation and activates gene expression [38]. Being closely interconnected, diverse epigenetic mechanisms mediate the precise pattern of gene expression during mammalian development, including fine-tuning of monoallelic expression and allelic imbalance.

One of the best-known examples of random allelic selection in mammalian somatic cells is X chromosome inactivation (XCI). This process results in approximately 50% of cells expressing paternal and 50% expressing maternal copies of genes on the X chromosome. In females, both X chromosomes remain active from the formation of the zygotic genome until the embryo attaches to the uterine wall. The first cytological evidence of single X chromosome inactivation (Xi) is observed in the late blastocyst to epiblast stages [39]. Although technically each X chromosome has an equal chance of being inactivated, in some cases deviations from a 50:50 ratio in inactivation patterns can occur. These scenarios include: (1) random skewing, (2) initial non-random selection for X chromosome inactivation due to the presence of specific variations in genes involved in the XCI process («primary» choice), or (3) positive or negative selection for cells carrying one particular active or inactive X chromosome due to the presence of specific variants in genes located on the X chromosome («secondary» choice) [40].

XCI is initiated at the X-Inactivation Center (XIC) and subsequently spreads across the future inactive X chromosome (Xi) during early embryogenesis. The central effector is the lncRNA *Xist*, which is expressed in an allele-specific manner from the XIC. Several chromatin modifying complexes are recruited during the initiation phase, including WTAP/Rbm15/Rbm15B, Polycomb repressive complexes 1 and 2 (PRC1 and PRC2), hnRNP U, and SMCHD1. Gene silencing is further established through extensive nuclear and genomic reorganization of the Xi, primarily characterized by repressive histone marks (e.g., H3K9me, H4K20me3) and Polycomb-mediated modifications (H3K27me3, H2AK119ub1), loss of H3K27ac, replacement of histone H2A by macrohistone H2A, DNA hypermethylation at CpG islands, and the reorganization of topologically associated domains (TADs) [41,42].

In contrast to the X inactivation, genomic imprinting is defined as an epigenetic mechanism which regulates gene expression based on the allele’s parental origin. In mammals, roughly 250 imprinted genes have been identified. These genes are mostly clustered together and controlled by cis-acting imprinting control regions (ICRs) [43]. Although genomic imprinting is crucial for gene expression regulation during the embryonic period, it also operates beyond development, influencing diverse tissues in adults; for instance, in the brain, it helps to regulate the survival and specialization of specific neuronal cells [44].

The main mechanism for allelic exclusion in the imprinted genomic regions is DNA methylation, although it is primarily guided by histone modifications. After the complete genome demethylation in primordial germ cells at the gastrula implantation stage, de novo methylation pattern is established in the ICRs to reflect the sex of the embryo [45]. In oocytes, the process of establishing DNA methylation at maternal ICRs takes place in the later stages of oogenesis (after birth but before ovulation) and begins with chromatin becoming permissive for DNA methyltransferases. This involves KDM1B removing H3K4me2 marks from intragenic CpG islands, while SETD2 deposits active H3K36me2 and H3K36me3 across transcribed regions. Paternal imprints are established during spermatogenesis in the adult male, specifically in prospermatogonia (primordial germ cells in the fetus) for some imprints, and finalized in postnatal spermatogonia undergoing differentiation. Unlike in oocytes, sperm methylation relies on the broadly distributed H3K36me2 mark, deposited by NSD1, rather than on H3K36me3. H3K36me2 directs DNMT3A and DNMT3L to methylate DNA at the paternal ICRs [46]. Thus, to date, no specific mechanism has been shown to direct de novo DNMTs specifically to imprinted loci, but the dependence of de novo DNMTs on histone methylation via H3K36 modification has been demonstrated in both oocytes and spermatozoa.

Apart from histone modifications, long non-coding RNAs are also known to contribute to the specific epigenetic landscape driving the establishment of canonical genomic imprints. In many ICRs, lncRNAs are transcribed from the active allele and mediate reciprocal silencing by recruiting chromatin remodeling factors and other enzymes. In the case of imprinted Kcnq1/Kcnq1ot1, a paternally expressed long noncoding RNA Kcnq1ot1 silences Kncq1 in trans, as well as several genes in cis. The lncRNA directly interacts with DNMT1, the maintenance DNA methyltransferase, to preserve DNA methylation at the ICR and downstream differentially methylated regions in somatic tissues. In parallel, Kcnq1ot1 recruits the components of PRC2 and the H3K9 methyltransferase G9A/EHMT2, which deposit repressive histone marks (H3K27me3, H3K9me3) and thus maintain the silent state of the paternal allele [47].

Although DNA methylation has always been thought to be an essential participant of the allelic exclusion in genomic imprinting, a different form of this epigenetic phenomenon known as noncanonical imprinting was shown to be driven by histone modifications, particularly by the interplay between H3K27me3 and H2AK119ub1 during oogenesis, and maintained in the absence of oocyte DNA methylation [48]. Maternal H3K27me3 is deposited by PRC2 on broad regions including the promoters of paternally expressed imprinted genes, whereas the paternal genome is largely protected from de novo H3K27me3 due to the presence of H3K36me3 on active genes. This repressive mark silences the maternal allele in the fertilized zygote, so that only the paternal copy is transcribed. After fertilization, the maternal H3K27me3 marks are retained through the blastocyst stage, as PRC2 recognizes the existing mark on the nucleosomes and copies it during DNA replication. The H2AK119ub1 mark, laid down earlier by PRC1, also helps to recruit PRC2 to reproduce the initial H3K27me3 landscape [48]. Importantly, post-implantation fate of noncanonical imprints is lineage-specific. Its maintenance in extraembryonic lineages is achieved through the de novo acquisition of secondary differentially methylated regions via Dnmt3a/3b and the H3K9 methyltransferase complex G9a/GLP. In embryonic lineages, however, noncanonical imprints are lost, resulting in epigenetic resetting prior to the onset of primordial germ cell development [46,48]. Thus, the establishment of the monoallelic expression pattern in non-canonically imprinted loci is mediated by histone modifications, but its maintenance and propagation still require DNA methylation.

Remarkably, all the described processes can deviate from strictly monoallelic expression patterns to a certain extent. A subset of X-linked genes have evolved strategies to evade silencing, allowing partially biallelic expression. Escape from XCI is often variable, occurring in an individual-, tissue-, or cell-type-specific manner [42]. The escape genes were shown to retain active histone marks such as H3K4me2, H3K9ac, H3K27ac, and H3K9me1, and lack the repressive mark H3K27me3 and the repressive histone variant macroH2A [49]. Moreover, the imbalance of histone marks measured by ChIP-seq correlates with the expression level: genes with higher Xi expression show lower imbalance values for the five active histone marks (H3K4me1, H3K4me3, H3K27ac, H3K27me3, H3K36me3), whereas genes that are strongly subject to XCI exhibit the highest imbalance of histone marks [50]. Likewise, a number of studies indicate that imprinted genes can be expressed biallelically or show a significant allelic bias [51,52,53,54]. DNA methylation studies indicate that several differentially methylated regions display inter-individual variability, as well as the methylation level of significantly above or below 50% [55,56]. The existence of the «leaky» or incomplete imprinting phenomenon underlies that epigenetic models tend to accommodate partial, tissue-specific, and inter-individual variation in the imprinting strength [53]. Supposedly, the same epigenetic mechanisms can cause RAExI observed in somatic tissues [33].

### 3.2. Histone Modifications and DNA Methylation in aRME

**Histone modifications and chromatin signature.** The massive combinatorial diversity of histone modifications enables complex regulation of gene expression. As shown in the previous chapter, the imbalance of allelic expression can be accompanied by the imbalance in the histone modifications associated with the corresponding alleles. One of the first attempts to identify a signature of histone modifications characteristic of random monoallelic expression was made in 2013. To investigate whether specific histone modifications characterize monoallelically expressed genes, Nag et al. compared chromatin marks between known MAE and biallelically expressed (BAE) genes by using data from the same cell type. The study showed that two different histone marks, the repressive modification H3K27me3 and the activating modification H3K36me3, are located in regions with monoallelically expressed genes, with this signature being characteristic for 20% of ubiquitously expressed genes and more than a third of tissue-specific genes [57,58]. Another study applied chromatin immunoprecipitation to screen for histone modifications in the promoter areas of monoallelically expressed genes [34]. It was demonstrated that H3K4me2 and H3K4me3 levels were increased in the clones with monoallelic and biallelic expression, consistent with the latter ones having twice the number of active alleles. Likewise, the H3K9me3 mark was found to be associated with repressed alleles. Intriguingly, bisulfite analysis of ten monoallelic genes showed no consistent allele-specific DNA methylation; most promoters were either unmethylated on both alleles or displayed methylation that did not correlate with allele activity. In attempts to establish the role of epigenetic regulators in maintaining allelic imbalance, Marion-Poll et al. showed that the silent allele is kept repressed by histone deacetylation; treatment with histone deacetylase inhibitors (Dacinostat, CUDC-101) increased the expression ratio of the previously silent *Bag3* allele, indicating that HDAC activity contributes to the maintenance of RAExI. The authors also analyzed ATAC-seq (assay for transposase-accessible chromatin) datasets in order to establish the regulatory regions controlling allelic imbalance. ATAC-seq data showed that allelic expression imbalance is tightly linked to allele-specific chromatin accessibility at the promoters of the studied RME genes. Linear regression between the allelic accessibility ratio obtained by ATAC-seq and the allelic expression ratio obtained by RNA-seq revealed a particularly significant correlation at the transcription start site; thus, RAExI is regulated locally at the promoter through allele-specific chromatin accessibility [33].

**DNA methylation.** Although random monoallelic expression seems to affect a subset of autosomal genes in various tissues, it is rather challenging to establish whether it can be maintained by random monoallelic DNA methylation. The main point of dispute lies in the question of how random DNA methylation can actually be, considering that by randomness we imply the absence of genetic variation as a driving force. After genomic imprinting caused by parent-specific mechanisms and random X inactivation, another well-characterized type of monoallelic DNA methylation is allele-specific DNA methylation (ASM) in which the level of CpG methylation strongly correlates with SNPs located nearby.

The ability of ASM to influence gene expression in the absence of other epigenetic factors has been discussed for a long period of time. Numerous studies indicate that genetic variation can often correlate with DNA methylation [59,60,61,62,63,64,65]. The first large-scale analysis of allele-specific DNA methylation was conducted in 2010 [3]. In this study, Schalkwyk et al. quantified ASM in 7.6% of the human genome (183,605 informative SNPs) using methylation-sensitive restriction enzyme (MSRE) and SNP microarrays in blood and buccal epithelial samples from monozygotic twins. The authors showed that ASM is a widespread phenomenon, potentially occurring at more than 35,000 sites across the genome, and that ASM is likely to be associated with heterogeneity between individuals.

Onuchic et al. generated a high-resolution atlas of allelic imbalance in DNA methylation, six histone marks, and transcription across 71 epigenomes from 36 cell and tissue types [66]. The study uncovered 4.9 M heterozygous loci, of which 5% exhibited >30% allele-specific CpG methylation. Allelic imbalance of epigenetic marks was shown to be enriched at enhancers and CpG-rich promoters, and for the majority of the ASM loci (71.7%), AI correlated with stochastic switching between fully methylated and unmethylated states for each of the two alleles. Moreover, it was demonstrated that heterozygous variants linked to ASM were enriched in genomic regions near variants previously associated with common diseases in genome-wide association studies (GWAS). The authors suggest that the convergence of GWAS signals and allelic imbalances within enhancers offers a mechanistic explanation for how allelic imbalance in DNA methylation may underlie GWAS associations. One of the latest studies examining the distribution of ASM across the genome was conducted by Rosenski et al. [67]. This work presents a comprehensive atlas of allele-specific DNA methylation obtained through deep whole-genome sequencing of 39 normal human cell types. 325,000 regions were identified, encompassing 6% of the genome and 11% of CpG sites, demonstrating a bimodal distribution of methylated and unmethylated sequences. SNPs that are inherited and exhibit allele-specific methylation were identified in 10% of the bimodal regions (34,000 loci). The authors identified 460 loci displaying allele-specific methylation inherited from parents, most of which were novel, as well as 78 loci associated with known imprinted genes. It was demonstrated that allele-specific, sequence- and parental-allele-dependent methylation is often restricted to certain cell types, revealing underappreciated variations in allele-specific methylation in humans. An interesting finding unveiled in the atlas was tissue-specific avoidance of imprinting. The presence of multiple, rarely studied cell types in the atlas revealed that many methylated loci associated with imprinting can escape monoallelic methylation and become fully methylated or completely unmethylated in specific cell types. The biological significance of this phenomenon and the underlying mechanisms likely vary depending on the gene and cell type.

Despite the acclaimed widespread distribution of ASM across the genome, the extent to which the genetic and epigenetic part of this interplay can affect gene expression level remains a subject of debate. A genetic variant may alter the DNA sequence of a local regulatory element, such as a promoter or enhancer, directly modifying the surrounding landscape for DNA methylation and thereby changing the accessibility of DNA to the transcriptional machinery. Alternatively, DNA methylation may occur as a consequence of the altered gene expression level due to the shift in the display of the transcriptional machinery.

In the 2013 study by Gutierrez-Arcelus et al., the issue of correlations between DNA methylation, genetic variation, gene expression and transcription factors’ abundance was addressed [68]. According to the data obtained by RNA-Seq, there are four models of interplay: (1) causal (active) pathway (a SNP first alters the methylation state of a CpG site; consequently, this change modulates gene expression level); (2) independent (passive) pathway (the same SNP independently affects both expression and DNA methylation, i.e., methylation level does not mediate the expression level); (3) expression-driven (passive) pathway (gene expression level changes first, and the altered transcriptional activity subsequently influences methylation levels); (4) transcription-factor (TF) mediation (TF abundance correlates with methylation at their binding sites, providing a mechanism for passive methylation changes driven by TF-level variation). The authors emphasize that the type of interaction between genetic and epigenetic factors depends on a cell type and genomic region. Another study proposed a model called «SNP intensifier» according to which the effect of a SNP is enhanced by the occurrence of monoallelic DNA methylation, which influences allelic recruitment or removal of TFs. Allele specificity of a TF ultimately increases or decreases the number of transcript copies from one allele, regulating the product dosage [69].

More recently, Stefansson et al. used Oxford Nanopore Technology long-read sequencing on 7179 whole blood samples to analyze haplotype-specific methylation at 15.3 million CpGs to investigate the correlation between CpG methylation and gene expression in the presence of in cis variants [70]. The study showed that most correlations between CpG methylation and gene expression are observed when methylation is linked to SNPs. Based on these results, Stefansson and co-authors proposed two models for the relationship between allele-specific methylation and altered gene expression. According to the first model, the allele characterized by SNP-specific methylation and altered gene expression levels alters the binding site of a TF. This change modifies TF binding, which in turn disrupts the local methylation pattern. However, it is the change in TF binding, not the change in methylation, that directly leads to the subsequent change in gene expression. In this scenario, methylation is a byproduct of the change in TF binding and does not play a causal role in transcriptional regulation. According to the second model, the specifically methylated allele also alters TF binding, but the resulting change in methylation has functional significance. In this case, methylation status influences gene expression, for example, by permitting or blocking the binding of a methylation-sensitive TF so that the epigenetic mark becomes an active transcriptional regulator. This scenario implies that DNA methylation is a mechanistic mediator between SNPs and expression. Thus, both models share the premise that genetic sequence variation is the primary driver of the observed correlation between methylation and expression levels.

Despite the fact that most experimental data indicate a passive role of DNA methylation in the regulation of gene expression, there is evidence of its active involvement outside the genetic context. A comprehensive analysis of 249 NPC clones, which has been mentioned earlier, established that both DNA methylation and histone modification take part in the regulation of monoallelic expression. Using *Bag3* as an example, it was shown that treatment with the DNA methyltransferase inhibitor (Decitabine), as well as two histone deacetylase inhibitors, significantly increase the ratio of expression of the silent allele [33]. The analysis of bisulfite-treated DNA from 20 NPC clones and ESCs demonstrated that the methylation level of CpG islands is high in silenced clones, low in biallelic clones and intermediate in monoallelic clones. This suggests that DNA methylation accumulates on low-expressed alleles during differentiation, correlating with transcript levels rather than with strictly mono- or biallelic expression. In consistency with these results, Gupta et al. established the «allele-specific rheostat» role for DNA methylation after utilizing the DNA-demethylating drug 5-aza-2′-deoxycytidine (5-aza-dC) [71]. Partial knockdown of the maintenance methyltransferase *Dnmt1* reactivated silenced alleles, establishing that Dnmt1-dependent methylation maintains allelic imbalance of expression for many MAE genes.

Overall, multiple lines of evidence suggest that DNA methylation, along with histone modifications, is at least one regulator of gene expression even in the absence of genetic variation. Although monoallelic DNA methylation not associated with SNPs appears to be uncommon in the genome, its role as a mechanism explaining phenotypic variability among genetically identical individuals cannot be ruled out. Therefore, both DNA methylation and the most characteristic histone marks can potentially be used to search for monoallelically expressed genes (Figure 2b). However, experimental data suggests that epigenetic marks should be considered indicative of clonal aRME with caution. Accurate identification of the genes truly subject to clonal aRME requires multi-layer profiling that includes genotyping, DNA methylation arrays, ChIP-seq, and allele-specific RNA-seq at the single-cell level and across multiple clonal populations. Only through such integrative approaches can the mechanisms responsible for the maintenance of monoallelic expression be reliably distinguished from transient transcriptional noise.

## 4. The Role of Monoallelic Expression in Pathological Processes

**Figure 3:**
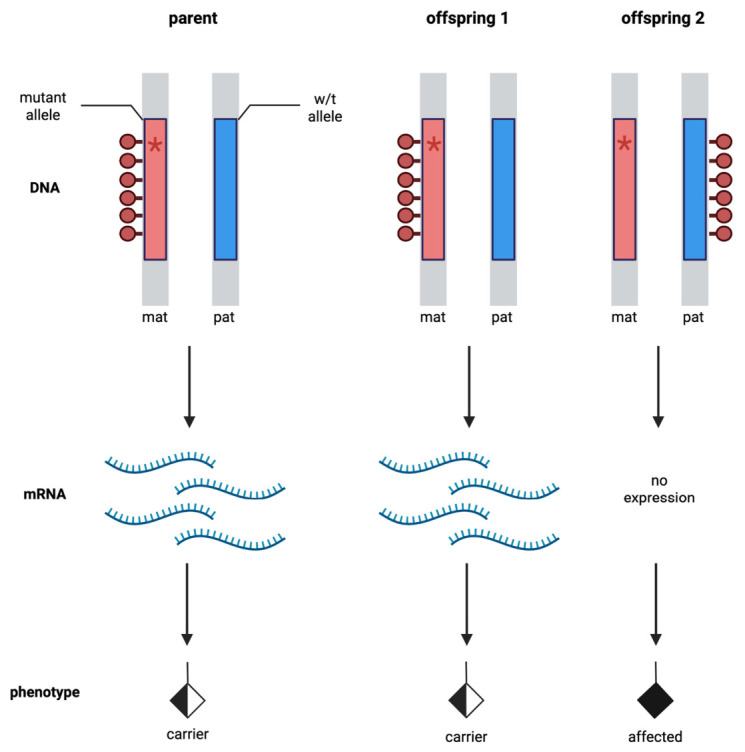
Random monoallelic expression can significantly influence the amount of the expressed product and, as a consequence, the severity of the clinical outcome in the presence of the same genetic variant within the same family. An asterisk represents genetic variant. mat, maternal allele (red-colored); pat, paternal allele (blue-colored). Created in https://BioRender.com.

The potential biological significance of autosomal monoallelic expression necessitates further research in this area. Variations in monoallelic gene expression play a crucial role in the interindividual differences in healthy individuals, but these changes can also have significant implications for the development of pathological conditions. As random monoallelic expression affects genes involved in many cellular processes, its occurrence may modulate the risk of the development of certain diseases. Random allele choice could potentially explain why identical pathogenic variants cause variable phenotypes: different cells or tissues may express either the mutant or the wild-type allele. Furthermore, random allele choice may result in a significant reduction in gene expression levels in the presence of a heterozygous pathogenic variant (Figure 3).

Gayan I. Balasooriya et al. studied the specific mechanisms of autosomal gene regulation in cardiac lineage cells, their significance, and possible impact on the development of heart and heart diseases. The authors proposed two types of monoallelic expression: deterministic monoallelic (DeMA), e.g., imprinted genes, and random autosomal monoallelic (RaMA) in which the allelic choice is not predetermined. They found that predisposition to heart disease depends on the expression of either the maternal or the paternal allele. When the critical gene is expressed only by the maternal allele, males are at a disadvantage in terms of disease penetrance, making them more susceptible to cardiovascular disease. The authors found that a group of DeMA genes with maternal expression in a cohort of cardiomyocytes is associated with a male-specific disease—3-methylglutaconic aciduria type 2 (Barth syndrome, MIM #302060). They presented genetic evidence that monoallelic genes with maternal expression could potentially be an etiological factor in heart disease in males. Meanwhile, genes with paternal expression demonstrated a predominant influence on cardiac physiology both in the neonatal period and in adulthood. This group of genes was regulated by the transcription factors (e.g., Esr1, SP1, Klf4) which are involved in the prevention of heart attack and myocardial hypertrophy. Clustering was observed for some genes in the DeMA group, which may contribute to the development of diseases caused by locus heterogeneity. The authors emphasized the importance of allele-specific gene expression for developmental processes, maintaining homeostasis, and the occurrence of cardiovascular diseases, and presented the results of identifying determinant monoallelic genes in specific cardiac cell types [72].

Fu R. et al. studied MAE during hematopoiesis and lymphomapoiesis at the single-cell level and identified a significant number of cases of stochastic monoallelic expression. This phenomenon, in which a single cell can stochastically express either of two alleles, was termed «biallelic mosaic rMAE». The authors observed higher levels of MAE in leukemic cells compared to normal cells, suggesting a link between MAE and leukemogenesis. Leukemia-specific MAE was established for the TCL6, TFDP2, and PTMA genes, which are associated with carcinogenesis and cell proliferation. A higher proportion of cells with MAEs for the PTMA gene were detected in a sample obtained at the relapse stage, suggesting that the PTMA gene status may predict relapse and poor prognosis in leukemia. The authors also identified enrichment of the histone modification H3K9me3 in leukemia cells at the relapse stage. Leukemia-associated MAE genes may serve as epigenetic therapeutic targets in the treatment of this disease [73].

Genes undergoing aRME have also been found to be frequently associated with autosomal dominant diseases in humans and haploinsufficiency in mice [32]. The authors observed that the proportion of cells in a given allelic state varied greatly in each differentiation experiment, which may contribute to incomplete penetrance of the disease associated with these genes between individuals. For example, the monoallelically expressed Bag3 gene is associated with autosomal dominant dilated cardiomyopathy with partial penetrance and has been described as haploinsufficient [74].

Similar data was obtained for the genes associated with autosomal dominant congenital immune disorders (CIDs)—genetic disorders that underlie susceptibility to infections, autoimmune diseases, autoinflammation, allergies, and/or malignancies. Stewart et al. studied the contribution of aRME to the phenotypic variability observed in families with CIDs. Using a clonal primary T-cell system to assess the aRME status of genes in healthy individuals, they found that 4.3% of genes responsible for the development of autosomal dominant CIDs and 5.2% of all genes are subject to aRME. The authors demonstrated that changes in allele expression are associated with H3K27me3 and DNA methylation, confirming previously proposed epigenetic mechanisms for aRME regulation. By studying aRME in peripheral blood mononuclear cells from individuals with CIDs, Stewart et al. demonstrated that in families with mutations in the PLCG2, JAK1, STAT1, and CARD11 genes, carriers with asymptomatic or mild disease symptoms often exhibit selective expression of the wild-type allele, whereas affected individuals with severe clinical manifestations exhibit biallelic or mutant allele expression. This study highlights the importance of considering not only the genotype but also the «transcriptotype» when analyzing the penetrance and expressivity of monogenic diseases [75].

MAE in specific tissues can also be important for assessing the increased risk of disease development, as it has been demonstrated for osteoarthritis (OA). Access to surgical specimens from OA patients has enabled analysis of allelic expression imbalance (AEI) in surgical specimens from various joint tissues. AEI analysis allows for the quantification of the relative ratio of mRNA transcripts produced by each allele of a given SNP. The authors studied the NCOA3 and SULF2 genes localized in the 20q13 region, which increase the OA risk and are associated with the rs6094710 SNP in patient cartilage cells. The NCOA3 and SULF2 genes encode nuclear receptor coactivator 3 and extracellular heparan sulfate-6-O-endosulfatase 2, respectively. NCOA3 expression is reduced in osteoarthritic cartilage compared to intact cartilage from the same joint, while SULF2 expression is increased in osteoarthritic cartilage compared to normal articular cartilage [76]. NCOA3 expression was shown to correlate with the genotype for rs6094710, and the gene exhibited AEI in individuals heterozygous for this SNP. The osteoarthritis risk allele (allele A) of this SNP was associated with reduced gene expression. Depletion of NCOA3 resulted in significant changes (all *p* < 0.05) in the expression of genes involved in cartilage homeostasis [77]. The study of OA risk associated with the T allele of rs4764133 revealed a decrease in matrix Gla protein (MGP) gene expression in cartilage compared to the non-risk C allele, confirming that this gene is the mediator of the OA risk [78]. A reduction in MGP (Matrix Gla Protein) expression was observed in other joint tissues, while the opposite effect was observed in whole blood samples, highlighting the tissue specificity of the molecular mechanisms underlying the disease risk and the biological pleiotropy exhibited by risk SNPs [79].

Several studies indicate that allele-specific expression (ASE) plays a crucial role in the initiation and progression of cancer. Niharika and colleagues investigated genome-wide ASE and monoallelic gene expression in non-small cell lung cancer (NSCLC). They analyzed 11,422 genes in metastatic NSCLC samples and found that roughly 43% (4935 genes) were undergoing monoallelic expression in at least one sample. On average, 21% of the genes showed monoallelic expression in each individual sample. The characterization of the genes has shown that they are involved in the realization of key signs of cancer, including tumor growth, metastasis, maintenance of the tumor microenvironment, and cell proliferation. This suggests that monoallelic expression plays an important role in the progression of NSCLC. The panel of monoallelically expressed genes includes a number of important NSCLC driver genes, such as CDH1, STK11, MYH9, ZNF395, TP53, EGFR, JAK2, RB1, CREBBP, FAT4, APC, CTNNB1 and TFRC. However, in addition to the driver genes, this gene panel was enriched with imprinted genes. Of the 4935 unique genes undergoing monoallelic expression, 52 genes were either already known imprinted genes or genes for which imprinting was predicted. MAE of different genes was observed in different samples, but 134 genes demonstrated stable monoallelic expression in all analyzed samples, which indicates that a significant proportion of MAE genes are individual in nature. In general, the data obtained can contribute to the development of effective diagnostic and prognostic models, and they can be used to develop personalized treatment strategies for NSCLC [80].

Silcock et al. examined the MAE prevalence, its frequency, and its dependence on damage in tumor-related genes in melanoma [81]. The authors examined 15 melanoma lines and found that the degree of MAE in melanoma ranges from about 17 to 77%, confirming its abundance. They did not show the dependence of MAE frequency on BRAF and NRAS mutations, as well as on the changes in the copy number of genomic loci. Comparing the levels of methylation between monoallelic and biallelic clones, the authors showed that MAE can only be partially explained by a change in DNA methylation. Thus, the phenomenon of MAE in tumor samples is quite well-established today, but there are still few studies investigating monoallelic expression in such tissues.

In another study, the authors investigated the relationship between the genes undergoing stochastic monoallelic expression (StMA genes) identified in clonal neural stem cells, and neurological disorders such as autism and schizophrenia [82]. Among the StMA genes, they found many candidate genes associated with the risk of developing schizophrenia, which were identified in genome-wide associative studies based on a database of genetic associations. In parallel, they examined multiple data on copy number variations (CNVs) in patients with autism and schizophrenia. The results indicate that the CNV regions associated with both autism and schizophrenia were also enriched with StMA genes compared to the control datasets. The authors suggest that random selection of expressed gene alleles in a cell population of a particular tissue may be a form of gene dose control. Additional effects are caused by deletions, duplications, or genetic regulatory variants that can directly affect allelic expression in the cell population and alter the dose of genes in the tissue [83]. It is assumed that StMA may contribute to a wide spread of cellular diversity during neurogenesis. Phenotypic diversity may also be enhanced due to polymorphisms in one or both alleles, or due to a decrease in the dose of the gene in cells with monoallelic expression, which may lead to the selective expression of risk alleles and increase the risk of the disease penetrance. Such a clonal distribution may also be a contributing factor to the discordance of disease phenotypes observed in monozygotic twins. Thus, the identification of StMA genes may also represent a unique way to identify new putative disease risk candidate genes.

## 5. Conclusions

The study of the molecular causes and biological significance of random monoallelic expression is still at an early stage. Our knowledge of the actual prevalence, stability, and functional consequences of this phenomenon remains highly fragmented. The technique that makes it possible to reliably determine stable monoallelic expression of genes in a single experiment has not yet been established, since it remains unclear whether there is a single mechanism responsible for monoallelic gene expression and whether it is universal for all types of tissues. In addition, one of the main obstacles in the study of monoallelic expression is the diversity of methodological approaches, which leads to the fact that studies that seem to focus on the same phenomenon often study fundamentally different processes. We have already mentioned a problem affecting the detection of monoallelic expression at the mRNA expression level: the lack of the uniform thresholds (such as the expression level or the AI ratio) leads to the fact that different research groups describe events characterized as aRME that are fundamentally different in their strength and reliability. However, a number of other methodological features also significantly affect our understanding of the prevalence and nature of aRME in the human genome.

The first and perhaps the most underestimated problem is related to the required number of cell clones under study. Clonal aRME is a feature that must be reproduced in genetically identical lines. The only reliable way to confirm that an allele-specific expression pattern is a stable feature of a cell, and not a random noise or artifact, is to analyze multiple clones grown from single cells. Ideally, the sample size should be tens or hundreds of clones, while current studies tend to draw conclusions about the presence of aRME based on the experiments involving 3–10 clones (Table 1). This number of clones makes it impossible to distinguish true monoallelic expression from stochastic events.

The second significant problem concerns the choice of biological material. The same genes may exhibit monoallelic expression in some tissues and biallelic in others. If random monoallelic expression is not caused by a cis-acting SNP, but is an exclusively epigenetic event, it usually presents with mosaic nature; therefore, it makes sense to study different types of tissues in order to predict the possible effect of the loss of a single expressed allele on the phenotype.

To characterize the events of truly monoallelic expression, it is also important to study the change in expression status over time during cell differentiation. This line of action is complicated by another problem, which is the extrapolation of data obtained on cultured cells to the in vivo state. Even when performed under strictly controlled conditions, cultivation is a stress-inducing event for the genome. It has been shown that cultivation with a small number of passages can provoke chromosomal instability [84]. It is known that DNA methylation levels in cell lines undergo a drift, and this drift is not accidental: some loci are systematically hypermethylated, while the methylation level of others decreases. Moreover, in vitro cultivation can induce the appearance of abnormal allele-specific patterns that are not present in the original tissue, and, conversely, neutralize stable in vivo patterns if they are supported by microenvironment signals (for example, paracrine factors or extracellular matrix) [85,86,87]. For these reasons, studies of clonal lines undergoing in vivo differentiation are of particular value for studying patterns of clonal monoallelic expression and the mechanisms of its distribution during cell division [25].

Despite the difficulties of studying monoallelic expression, it is of great clinical significance to map such loci. The list of diseases the course of which can be influenced by monoallelic expression continues to expand. Knowledge on the distribution of genes undergoing aRME in the human genome would make it possible to predict the phenotypic manifestations of hereditary diseases much more accurately, since with accidental inactivation or activation of one of the alleles, identical genotypes can lead to different clinical outcomes, from asymptomatic carriage to severe pathology. Considering monoallelic expression patterns along with traditional genotyping will make it possible to personalize risk assessment and prognosis of the course of monogenic and multifactorial diseases.

## Figures and Tables

**Table 2:** Histone modifications and their effect on gene expression.

Modification	Effect on Transcription	Modifiable Histone Sites
Acetylation	Activation	H3 (K9, K14, K18, K56); H4 (K5, K8, K12, K16); H2A/H2B (K6, K7, K16, K17)
Phosphorylation	Activation	H3 (S10)
Methylation	Activation	H3 (K4, K36, K79)
	Repression	H3 (K9, K27)
Ubiquitination	Activation	H2B (K123)
	Repression	H2A (K119)
Sumoylation	Repression	H4 (K5, K8, K12, K16), H2A (K126), H2B (K6, K7, K16, K17)

## Data Availability

No new data were created or analyzed in this study. Data sharing is not applicable to this article.

## Abbreviations

The following abbreviations are used in this manuscript:

AEI/AI	Allelic expression imbalance/allelic imbalance
aRME	Autosomal random monoallelic expression
ASM	Allele-specific methylation
BS-seq	Bisulfite sequencing
ChIP-seq	Chromatin immunoprecipitation sequencing
CIDs	Congenital immune disorders
CNS	Central nervous system
CpG	Cytosine-phosphate-guanine dinucleotide
DeMA	Deterministic monoallelic expression
DNA	Deoxyribonucleic acid
DNMT	DNA methyltransferase
ESC	Embryonic stem cell
FACS	Fluorescence-activated cell sorting
GWAS	Genome-wide association studies
HSC	Hematopoietic stem cell
ICR	Imprinting control region
MAE	Monoallelic expression
NPC	Neural progenitor cell
NSC	Neural stem sell
NSCLC	Non-small cell lung cancer
OA	Osteoarthritis
RaMA	Random autosomal monoallelic expression
RAExI	Random allelic expression imbalance
RNA	Ribonucleic acid
scRNA-seq	Single-cell RNA sequencing
SNV/SNP	Single-nucleotide variant/polymorphism
StMA	Stochastic monoallelic (expression)
TF	Transcription factor
XCI	X chromosome inactivation
